# Post-COVID-19 Epidemiology of Viral Infections in Adults Hospitalized with Acute Respiratory Syndromes in Palermo, South of Italy

**DOI:** 10.3390/pathogens14100997

**Published:** 2025-10-02

**Authors:** Mariangela Pizzo, Floriana Bonura, Federica Cacioppo, Emilia Palazzotto, Chiara Filizzolo, Sharon Russo, Daniela Pistoia, Giuseppina Capra, Donatella Ferraro, Giovanni M. Giammanco, Simona De Grazia

**Affiliations:** 1 Department of Health Promotion, Mother and Child Care, Internal Medicine, Medical Specialties (PROMISE), University of Palermo, 90127 Palermo, Italy; mariangela.pizzo01@unipa.it (M.P.); giovanni.giammanco@unipa.it (G.M.G.); simona.degrazia@unipa.it (S.D.G.); 2 Microbiology and Virology Unit, University Hospital “Paolo Giaccone”, 90127 Palermo, Italy

**Keywords:** respiratory viruses, respiratory tract infections, syndromic assays, COVID-19 pandemic, seasonality, Italy

## Abstract

This study evaluated the epidemiology and seasonal patterns of respiratory viruses in adults hospitalized with acute respiratory tract infections during two consecutive post-COVID-19 pandemic seasons. A retrospective study was conducted at the University Hospital “P. Giaccone”, Palermo, from September 2022 to September 2024. Multiplex molecular assays were used to detect the ten respiratory viruses most relevant from an epidemiological perspective in respiratory samples (n = 1110) of 1081 patients. A respiratory viral infection was identified in 29.6% of patients. The highest viral infection rate was observed in the 31–50 age group. Human rhinovirus/enterovirus (HRV/EV) was the most frequently detected (40%), followed by influenza A virus (IAV; 18.4%) and human coronaviruses (HuCoVs; 12.8%). Viral co-infections were identified in 10.9% of positive cases, with HRV/EV, adenovirus (ADV), and parainfluenza virus (PIV) being most frequently involved. Influenza and respiratory syncytial viruses (RSVs) showed a winter seasonality, while diverse circulation patterns were revealed for the other viruses. This study demonstrated a sustained circulation of respiratory viruses in adults hospitalized with severe respiratory symptoms, with HRV/EV accounting for most of them. Syndromic multiplex molecular testing, although limited to the detection of a small fraction of epidemiologically relevant known viruses, has proven to be a valuable tool, not only for diagnostic purposes but also for acquiring genotyping data and implementing epidemiological information from sentinel surveillance systems.

## 1. Introduction

Human respiratory tract infections (RTIs) represent a significant global public health concern, being associated with high mortality and morbidity rates, as well as substantial economic losses. These infections can affect both the upper and lower respiratory tract. Although most upper respiratory tract infections (URTIs) are typically self-limiting and mild, some can lead to severe complications. These include progression to lower respiratory tract infections (LRTIs), such as pneumonia and acute bronchitis, as well as acute exacerbations of chronic obstructive pulmonary disease (COPD), worsening of bronchiectasis, and even myocarditis [1]. Vulnerable people, including children, the elderly (aged 65 and above), and immunocompromised patients, are particularly at risk for these outcomes [2]. The etiology of RTIs encompasses various pathogens, including viruses, bacteria, and fungi, which often present with overlapping symptoms, making clinical differentiation challenging. Discerning viral infections from bacterial or fungal ones is paramount to avoid inappropriate antimicrobial therapies and for the successful management of inpatients [3]. Among viral infections, respiratory viruses, such as influenza viruses A and B (IAV and IBV); respiratory syncytial virus (RSV); parainfluenza virus (PIV); adenovirus (ADV); and human coronavirus (HuCoV), belong to diverse virus families that differ in their morphology, genomic characteristics, susceptible host, disease severity, seasonality of circulation, and transmissibility [4]. Generally, non-enveloped viruses exhibit greater resilience to environmental conditions, such as extreme pH, heat, desiccation, and basic disinfectants, than enveloped ones, contributing to their enhanced ability to maintain infectivity over extended periods [5,6]. Despite such variability, a high infectivity rate and short incubation period are common traits among all respiratory viruses, contributing to their rapid spread and the occurrence of outbreaks [7]. In temperate regions, respiratory viruses follow distinct seasonal patterns, with influenza, RSV, and HuCoVs peaking in winter, while ADV and rhinovirus (HRV) circulate year-round, and some enteroviruses (EVs) show increased prevalence during the summer [8,9]. Differences in viral transmissibility reflect variations in epidemiological patterns. The basic (R_0_) and effective (R_e_) reproduction numbers are key epidemiological indices for evaluating viral transmissibility over time and under varying population immunity conditions. In particular, R_0_ quantifies the average number of secondary infections generated by a single infectious individual in a population without immunity and interventions. An R_0_ > 1 indicates that the virus is spreading within the population, with epidemic or pandemic potential, whereas values of R_0_ < 1 suggest declining transmission and eventual containment [10,11]. Conversely, the effective reproduction number (R_e_) accounts for the level of a population’s immunity, acquired through infection or vaccination, as well as biological, behavioral, and environmental factors, providing a more accurate estimate of viral transmission at a given time. Thompson et al. showed the median R_e_ of influenza A and B was 1.23, comparable to previous estimates for seasonal influenza [12]. The severe acute respiratory syndrome coronavirus 2 (SARS-CoV-2 or COVID-19) pandemic had a profound impact on global respiratory health, leading to unprecedentedly high case and death counts, with a high epidemic growth rate and an estimated value of R_0_ for the first variant of SARS-CoV-2 ranging from 3.6 to 6.1 in European countries and 5.8 in the United States [10]. Several non-pharmaceutical interventions (NPIs), including social distancing, mask-wearing, hand hygiene, travel restrictions, school closures, and vaccination, introduced to contain the spread of COVID-19, have influenced the evolution of the virus, driving the emergence of SARS-CoV-2 variants with different levels of transmissibility and adaptability to human hosts. The average R_e_ for the Omicron variant has been estimated at 3.4 with a range of 0.88 to 9.4 [13]. Moreover, the SARS-CoV-2 pandemic has changed the dynamics of respiratory viral transmission, altering the typical seasonal circulation patterns of other respiratory viruses, particularly those of enveloped viruses, through the impact of non-pharmaceutical interventions, as well as viral interactions such as competition, co-infection, and immune interference [6,14,15]. A key lesson learned from the COVID-19 pandemic has been the importance of the rapid and accurate diagnosis of RTIs to ensure timely and appropriate treatment, reduce transmission, monitor viral prevalence and emerging strains, and minimize unnecessary antibiotic use [3]. Advances in syndromic molecular diagnostic tools, which offer enhanced sensitivity, may have the potential to reshape current approaches to the diagnosis and management of RTIs [16,17]. This study aimed to investigate the epidemiology of respiratory viral pathogens among adult patients hospitalized with RTIs during two consecutive epidemic seasons post-COVID pandemic (2022/23 and 2023/24) at the University Hospital of Palermo.

## 2. Materials and Methods

### 2.1. Study Design

This study aimed to evaluate changes in the epidemiological features of RTIs among adults hospitalized with severe respiratory symptoms in the post-COVID-19 pandemic context. A retrospective, observational, single-center study was conducted using diagnostic assay results routinely collected at the virology laboratory of the University Hospital “P. Giaccone” in Palermo, Italy.

### 2.2. Study Population and Clinical Samples

From September 2022 to September 2024, a total of 1110 respiratory samples, including 792 upper respiratory tract specimens (nasopharyngeal swab or nasal swab, NPS) and 318 lower respiratory tract samples (bronchoalveolar lavage or sputum, BAL), were investigated for diagnostic purposes using syndromic assays for viral infections. The respiratory specimens were obtained from 1081 adults (>18 years) hospitalized with acute respiratory symptoms, including fever (38 °C), cough, dyspnea, wheezing, lung auscultation abnormalities, tachypnoea, and chest pain. From September 2022 to March 2023, only patients who tested negative for SARS-CoV-2 were screened (n = 287/1081), whereas afterward, the patients (n = 794/1081) were enrolled regardless of SARS-CoV-2 status, and all samples were analyzed using either SARS-CoV-2-specific molecular assays combined with respiratory panels for other viruses or multiplex panels that included SARS-CoV-2 detection (Figure S1). Patient demographics (gender and age) and clinical data (symptoms and ward of admission) were extracted from anonymized medical records and subjected to statistical analysis. Positive cases of viral infection were classified as community-acquired pneumonia (CAP) when respiratory manifestations suggestive of pneumonia were present upon hospital admission. Conversely, patients who did not exhibit respiratory-related signs at admission or who underwent respiratory virus testing for the first time more than 48 h after hospitalization were defined as having hospital-acquired pneumonia (HAP). Based on age ranges, patients were stratified into 8 different groups: 18–30, 31–40, 41–50, 51–60, 61–70, 71–80, 81–90, and over 90 years.

### 2.3. Microbial Pathogen Detection

The differential diagnosis of viral RTIs in patients included in the study was performed using several commercial syndromic molecular tools. Specifically, for the detection of viral etiological agents of pneumonia, the following commercial tests were used: Seegene RV Master Assay and Allplex™ Respiratory Panels 1–3 (Seegene Inc., Songpa-gu, Seoul, Republic of Korea) and BIOFIRE FilmArray Pneumonia plus [FAPP] and FilmArray Respiratory Panel 2.1 [RP2.1] (BioFire Diagnostics, Salt Lake City, UT, USA). These assays differed in terms of the number of detectable targets, performance characteristics, and turnaround time. For the completeness of the screening for SARS-CoV-2 infection, the Xpert^®^ Xpress SARS-CoV-2/Flu/RSV test (Cepheid, Sunnyvale, CA, USA) and the Simplexa COVID-19 Direct assay (DiaSorin Molecular, Cypress, CA, USA) were also used. Notably, human bocavirus (HuBoV) is included as a detectable target only in Allplex Respiratory Panel 3. However, for the sake of completeness of viral prevalence estimates, HuBoV-positive cases were included in the overall analysis; statistical comparisons were performed separately on the subgroup of patients tested. The patients were diagnosed as positive for viral infection if they had a positive result in at least one of these tests. All syndromic molecular tests were performed according to the manufacturer’s instructions. Specifically, a total of 1120 syndromic molecular tests were performed, including 10 samples tested by two different molecular tools to include SARS-CoV-2 in the range of detectable pathogens. Table S1 details the number of assays conducted on NPS and BAL samples, categorized according to origin from intensive care units (ICUs) or non-intensive care units (non-ICUs), the latter including departments such as general medicine, respiratory medicine, infectious diseases, geriatrics, or other hospital specialties

Among the study population, 834 patients tested for viral infections were also screened for bacterial and fungal pathogens using standard sputum culture and/or BIOFIRE FilmArray Pneumonia Panel Plus, which can identify 15 typical bacteria, 3 atypical bacteria, and 7 antimicrobial resistance (AMR) genes. For *Chlamydia pneumoniae*, *Mycoplasma pneumoniae*, and *Legionella pneumophila*, serological tests were also used

### 2.4. Statistical Analysis

Pearson’s, Chi-square, or Fisher’s exact tests were used to compare the category variables between groups when appropriate. The virus detection rate was calculated by dividing the positive cases by the total number of tested patients. All statistical analyses were performed using Excel (Microsoft 2018) and R version 4.2.3 (R Foundation for Statistical Computing, Vienna, Austria). Statistical significance was defined as a *p*-value of <0.05.

## 3. Results

### 3.1. Patient Characteristics

During the two-year study, a total of 1081 adults hospitalized for RTIs were investigated by multiplex Polymerase Chain Reaction (PCR) assay, including 539 patients tested in the first post-COVID-19 pandemic season (September 2022 to August 2023) and 542 in the second (September 2023 to September 2024). The male-to-female ratio was 1.30 (with 56.5% males), and the median age of recruited patients was 71 years (interquartile range [IQR]: 58–79). The demographic information and admission wards of the patients are reported in Table 1.

Overall, a viral infection was detected in 320/1081 (29.6%) patients. Specifically, among the 1110 samples analyzed from the study population, 81/318 (25.5%) BAL and 250/792 (31.6%) NPS samples were positive for at least one virus (OR = 0.74, 95% CI: 0.55–0.99; *p* = 0.053). No statistically significant difference in positivity rates was observed between male and female patients (*p* = 0.297). According to the criteria used to differentiate nosocomial infections from community-acquired pneumoniae, 223/320 positive patients (69.7%) were classified as having CAP, while the remaining 97 (30.3%) were classified as having HAP. Among the 223 patients with CAP, a viral infection was detected in 34 BAL (15.2%) and 182 NPS (81.6%) samples, and in 7 cases (3.1%), a viral infection was detected in both high and low respiratory tract specimens. Conversely, HAP infections were diagnosed by the detection of at least one virus in 36 BAL samples (37.1%), 57 NPS samples (58.8%), and in both specimen types in four cases (4.6%). Age stratification revealed a statistically significant difference between age groups (χ^2^ = 17.5, *p* = 0.014). The highest viral infection rates were observed in the 31–40 (46.3%, 19/41) and 41–50 (37.0%, 27/73) age groups and the lowest rate in the 71–80 group (22.3%, 75/336), while intermediate and comparable rates were observed among other age groups: 28.3% (13/46) in the 18–30 group, 30.5% (46/151) in the 51–60 group, 32.4% (71/219) in the 61–70 group, 32.0% (62/194) in the 81–90 group, and 33.3% (7/21) in patients over 90 years. Further statistical analysis using Pearson’s χ^2^ test revealed a slight negative correlation between age and the proportion of patients testing positive for viral infections (r = −0.33). No significant difference was observed in median age (68 vs. 68.5) between CAP and HAP patients. However, a higher viral CAP proportion was seen among young adults, with 31.7% in the 31–40 age group and 28.3% in the 18–30 age group. Instead, HAP percentages were constant across different age groups, ranging from 8.0% to 14.6%.

### 3.2. Detection of Respiratory Viruses

Among patients with a viral infection, 285 out of 320 (89.1%) were positive for a single pathogen, while two or more targets were found in 35 cases (10.9%). With regard to Picornaviridae, the BIOFIRE FilmArray assay could not distinguish HRV and EV positivity and generated a result indicating “Human Rhinovirus/Enterovirus detected” in 28 out of 308 patients (9.1%). Conversely, the Allplex Respiratory Panels allowed us to distinguish HRV infections in 92 (11.8%) cases and EV infections in 8 (1.0%) out of 778 patients. To simplify the analysis, all positive results for HRV/EV (Filmarray) and HRV or EV (Allplex) were considered together, as a single pathogen referred to as HRV/EV, though we are aware that the vast majority should be ascribed to HRV infections. Taking into account the previously discussed limitations, the most commonly identified virus among the patients with a viral infection in our study was HRV/EV (40%, 128/320), followed by IAV (18.4%, 59/320) and HuCoVs (12.8%, 41/320). In particular, in CAP cases, HRV/EV had the highest positive rate (40.3%; 90/223), followed by IAV (20.2%; 45/223), whereas HAP, HRV/EV, and HuCoVs were the most prevalent viral pathogens (39.2%, 38/97, and 18.5%, 18/97, respectively). The overall viral infection rate increased slightly from 27.4% (148/539) in the 2022–2023 season to 31.7% (172/542) in 2023–2024, but no statistically significant difference was observed between the two seasons (*p* = 0.141). Several pathogens showed a decline in detection rates when comparing the 2022–2023 and 2023–2024 seasons: IAV decreased from 22.3% to 15.1%, RSV from 10.8% to 8.7%, Metapneumovirus (MPV) from 9.5% to 4.1%, and IBV from 2.7% to 1.2%. Conversely, other viruses showed increased prevalence in the second season. HuCoVs rose from 5.4% to 19.2%, PIV from 4.7% to 11.0%, and HuBoV from 1% to 4.6%, while HRV/EV and ADV prevalence remained stable in both seasons (40.5% vs. 39.5% and 6.8% vs. 7.6%, respectively). SARS-CoV-2 was included in the analyses only beginning in March 2023, reaching 4.7% prevalence in the last six months of the first season and increasing to 8.1% in the following season. Regarding the age distribution of viral infections, a significant association (*p* < 0.05) was obtained for HRV/EV infections in the 31–40 group, for PIV in the 81–90 group, and for IBV in the 18–30 group. Furthermore, associations close to the 0.05 *p*-value significance threshold were identified for HRV/EV in the 71–80 group, IBV in the 41–50 group, and ADV in the 31–40 group. Conversely, no statistically significant age-based patterns were shown for the other viral pathogens (Table S2). As shown in Table 2, the comparison of the virus distribution between ICU and non-ICU patients revealed that HRV/EV, RSV, and ADV were significantly more frequent in non-ICU patients, while HuCoVs were the only pathogens whose detection rate was higher among ICU patients (6.2% vs. 3.1%). Furthermore, HuCoVs were detected significantly more often in BAL fluid samples compared to NPS samples (6.9% vs. 2.4%, *p* < 0.05), whereas ADV was more commonly isolated from NPS specimens (0.6% vs. 2.6%, *p* < 0.05). All other viruses showed no statistically significant differences in detection rates between the two types of specimens (Table 2).

**Figure 1:**
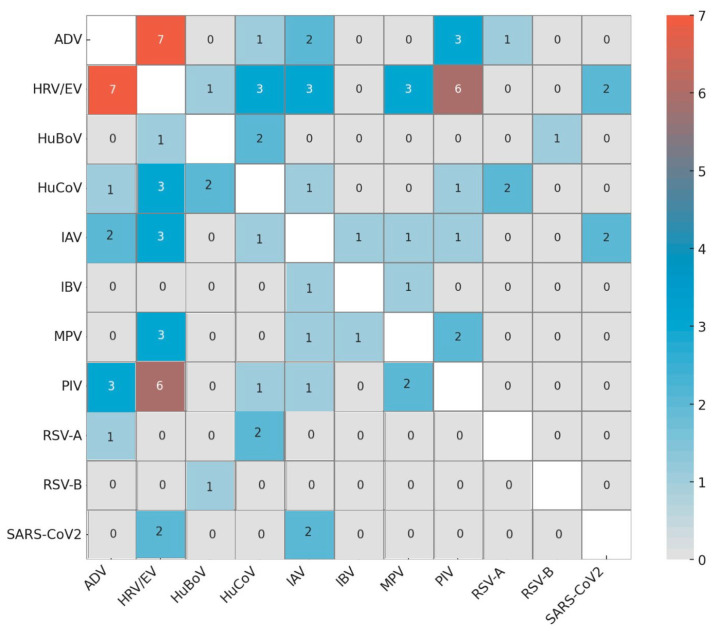
Frequency of virus combinations. Heatmap of co-infection frequencies between virus pairs.

Among the 35 co-infections involving viruses detectable by the syndromic panels used, two or more targets were detected in 27 (10.8%) NPS-positive and 8 (9.9%) BAL-positive samples. Furthermore, 57.1% of subjects with viral co-infections were aged over 65 years. Comparison of Ct values (threshold cycle), used as a proxy for viral load, was available for 31 of 35, as 4 samples were tested by the BioFire platform, which provides only qualitative results without Ct values. The predominance of one virus over the others, calculated comparing the Ct values, was detected in 19 of 31 (61.3%) of the co-infections, with an average Ct difference of 13.6 (Ct average being 22.9 for the most represented target and 36.6 for the least represented), while in 12 (38.7%) virus–virus associations, no viral predominance was detected, and the average Ct values were similar (34.6 vs. 36.3). The frequency of viral combinations in co-infections is shown in Figure 1.

The Ct values of pathogens detected in co-infections are shown in Table S3. The distribution of each virus in mono- or co-infections revealed that the majority of viruses were significantly associated with mono-infections, with HuBoV being the virus most frequently detected in association with other viruses (42.9% mono-infections vs. 57.1% co-infections), albeit lacking statistical significance (*p* > 0.05) (Table S4).

### 3.3. Virus Seasonality

**Figure 2:**
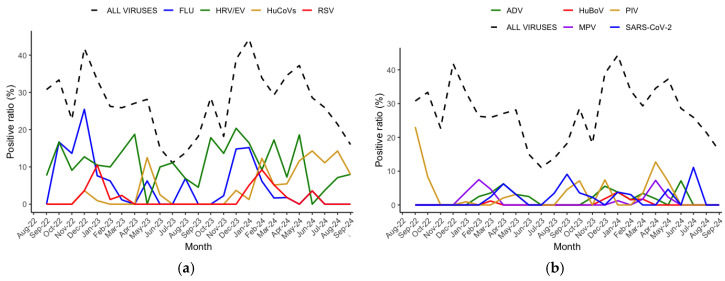
(**a**) Seasonal distribution of viruses with a prevalence ≥9%, including HRV/EV, influenza (FLU), RSV, and HuCoVs; (**b**) seasonal distribution of viruses with a prevalence <9%, including PIV1/2/3/4, MPV, ADV, HuBoV, and SARS-CoV-2. On the X-axis, the months of the study period are shown; on the Y-axis, the monthly positivity rate of each virus is reported. In both figures (**a**,**b**), a dotted line indicating the overall prevalence of viral infections is shown.

In the two epidemic seasons, peaks of viral infections occurred in the winter months, with the highest positive rates occurring in December and January in both seasons investigated. The seasonality patterns of viruses detected are shown in Figure 2a,b. In particular, during the two post-COVID-19 pandemic seasons, influenza peaks were observed starting in October 2022 and December 2023. In contrast, HRV/EV showed persistent levels of detection throughout the study period.

### 3.4. Virus Genotyping

**Figure 3:**
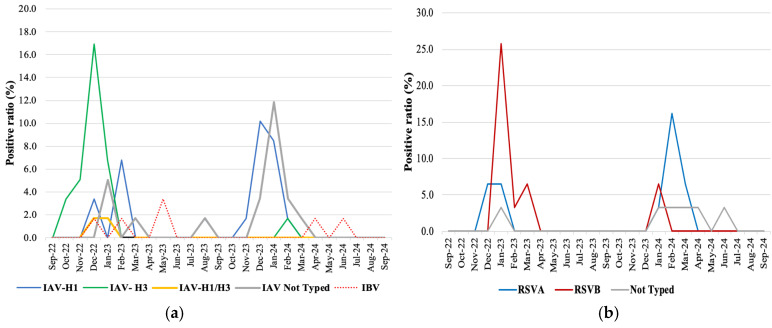

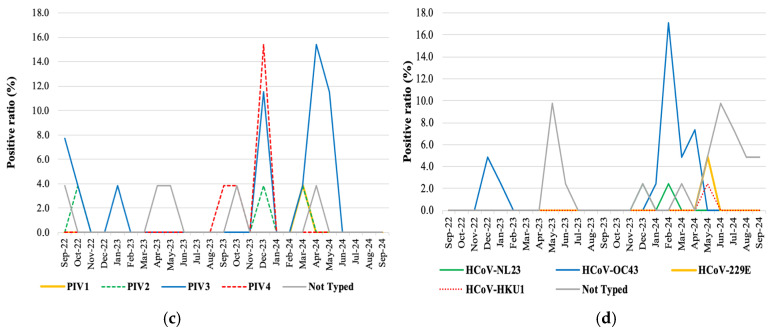
(**a**) Seasonal incidence of IAV and IBV; (**b**) RSVs A-B; (**c**) PIVs 1–4; and (**d**) HuCoVs.

Genotyping data, provided by Allplex Respiratory Panels 1–3, were available for 73.8% (48/65) of influenza viruses detected, 80.6% (25/31) of RSVs, 80.8% (21/26) of PIVs, and 51.2% (21/41) of HuCoV-positive cases. In particular, molecular characterization revealed the predominance of the influenza type A/H3 in the 2022–2023 season (20/33, 60.6%), while in the 2023–2024 season, A/H1 was prevalent (20/26, 76.9%), with 75% (15/20) of detections being subtype A/H1N1pdm09 (Figure 3). Furthermore, in two influenza-positive samples, the simultaneous presence of the A/H3 and A/H1 types, with comparable Ct values, was observed. Similarly, RSV type B was the predominant genotype in the first season (11/16, 68.7%) and was replaced by RSV-A in the following season (8/15, 53.3%), as shown in Figure 3b. Of the PIVs detected, PIV3 was the predominant genotype (15/26, 57.7%), with peaks of circulation in December 2023 (11.5%), April (15.4%), and May (11.5%) 2024, followed by PIV 4, detected in 2023 in September (3.8%), October (3.8%), and December (15.4%), while PIV1 and PIV2 were detected only sporadically (Figure 3c). Among HuCoVs, HuCoV-OC43 accounted for 39.2% of detections, mainly during the winter (Figure 3d).

### 3.5. Non-Viral Pathogen Detection

Among the 1081 patients tested for viral infections, data on bacterial and fungal infections were available for 834 individuals. Of these, 52.2% (435/834) tested positive for bacterial or fungal pathogens, and mixed infections involving both viral and non-viral agents were identified in 25.9% (113/435) of the positive cases.

No significant sex-related differences were observed in the overall positivity rate for respiratory infections, whether viral or non-viral (χ^2^ = 0.084, *p* = 0.77).

Regarding age distribution, the prevalence of non-viral infections progressively increased with advancing age in male patients, particularly from age 50 onward, reaching the highest prevalence in the 71–80 age group (71/160; 44%). However, a 60.9% positivity rate for bacterial infections was also recorded among the 23 patients in the 18–40 age group. Mixed infections were observed across all age groups, with the highest prevalence in patients aged 41–50 years (35.5%), followed by those aged 61–70 years (16%). Conversely, a higher prevalence of non-viral infections (51.4%) was observed among females aged 41–50 years (Table 3).

Notably, 54.8% of the 435 patients with non-viral infections required ICU admission, underscoring the potential severity of these infections. Furthermore, 13% of these ICU patients presented with mixed viral and non-viral infections.

## 4. Discussion

The SARS-CoV-2 pandemic has profoundly influenced the epidemiological patterns of common respiratory viruses, mainly due to the effect of NPI implementation, which has reduced opportunities for viral transmission [15]. In addition, experimental and observational studies suggest that virus/pathogen interference mechanisms and non-specific innate immune responses, such as those mediated by type I and III interferons, may also have contributed to the changes in pathogen balance observed during the COVID-19 and post-COVID-19 periods, highlighting the critical role of viral interaction in shaping epidemic dynamics [14]. However, these effects remain difficult to ascertain, principally due to the underdiagnosis of other respiratory viruses during the pandemic [18,19,20]. The emergence of COVID-19 highlighted the critical need for new and more effective tools to support the differential diagnosis of RTIs [15,21,22,23]. To address these diagnostic challenges, commercial syndromic tests enabling the simultaneous detection of multiple respiratory pathogens should be used [24]. Moreover, many syndromic assays provide valuable data allowing for deeper insights into the circulation of respiratory virus genotypes [25]. The post-COVID-19 period, defined by the relaxation of NPIs and increased vaccination coverage for SARS-CoV-2, represents a unique and unprecedented context for analysis, characterized by epidemiological conditions and dynamics that are unlikely to occur again. In this study, different multiplex molecular panels, with comparable sensitivity and specificity, were employed to investigate the prevalence of respiratory viral infections among adults hospitalized with severe respiratory symptoms during two consecutive post-COVID-19 pandemic seasons (2022–2023 and 2023–2024) in Palermo, southern Italy. A viral respiratory infection was identified in 29.6% of the tested population and was associated with the development of CAP in approximately two-thirds of the positive cases (69.7%), while the remaining 30.3% were classified as HAP. A higher proportion of viral infections was found in the younger–intermediate age groups (31–50 years), but no significant differences were observed between males and females (28.3% and 31.3%). National and European epidemiological surveillance data for influenza-like illnesses (RespiVirNet and ERVISS) showed a higher incidence of influenza-like syndromes in the 15–64 age group compared to older populations in the same seasons [26,27]. Moreover, similar trends in the age distribution of viral infection rates have been reported in previous national and international studies focusing on adult hospitalized patients during both pre-COVID-19 pandemic and COVID-19 pandemic periods [28,29]. More frequent social interactions or occupational exposures may facilitate viral transmission among young people, although this population should be less prone to serious infections requiring hospitalization [30]. In addition, age-related differences in gene expression of cellular components involved in viral adsorption; entry; RNA release and replication; progeny particle assembly; and viral dispersal have been documented. These age-related variations in susceptibility to viral infections and immune responses are reflected in the distinct mortality profiles observed in different variants of COVID-19. In particular, the Delta variant disproportionately affected individuals under the age of 65, while the Wuhan and Alpha variants were associated with a relatively high percentage of deaths among individuals under the age of 84. In contrast, the Omicron variant, although characterized by a substantially lower overall mortality rate, showed a relative shift toward older age groups, with a progressive increase in mortality after age 65 and reaching the highest levels among centenarians [31]. The results of this study contribute to increasing knowledge on the impact of viral agents on RTIs among adults, a population in which bacterial etiologies are generally more prevalent. This contrasts with neonates, for whom the burden of hospitalization due to viral respiratory infections is well documented in the literature [32,33,34]. In our cohort, data on non-viral infections were available for 77.1% of patients, revealing a positivity rate of 52.2% (435/834), including 25.9% (113/435) of mixed infections. It is well established that previous or concomitant viral respiratory infections compromise airway integrity and innate immune defenses, thereby strongly predisposing the host to secondary bacterial pneumonia, a complication strongly associated with worsening clinical outcomes [35]. Notably, a progressive increase in non-viral infection detection was observed in patients over 50 years of age. Among those with non-viral infections, 54.8% required admission to intensive care units, underscoring the association between non-viral respiratory infections and severe clinical outcomes. Overall, 34.2% of patients tested negative for the specific and restricted spectrum of infectious agents examined by the syndrome panels used. The accuracy of respiratory pathogen detection is closely linked to the choice of biological specimen, which is particularly critical in virological investigations. Both BAL and NPS samples are commonly used for respiratory pathogen detection and are preferred depending on the clinical context. In particular, BAL represents the ideal specimen to diagnose LRTIs, but, as it requires invasive bronchoscopy procedures, it is most commonly used for the diagnosis of severe pneumonia. Conversely, NPS samples are generally used for routine screening of URTIs, but they suffer from operator-dependent variability in the sampling quality [36,37]. In our study, most of the BAL samples (233/318, 73.3%) were retrieved from patients admitted to the ICU, while NPS was the prevalent specimen in non-ICU wards (776/792, 97.9%). Despite these differences in clinical setting and specimen type, no significant difference in viral positivity rates was observed between BAL (25.5%) and NPS (31.6%), suggesting comparable diagnostic yield in this context.

During the study period, characterized by the emergence and widespread circulation of the Omicron variant of SARS-CoV-2, HRV/EV was the most frequently identified virus in both community-acquired pneumonia (CAP) and hospital-acquired pneumonia (HAP) cases, with stable levels of detection prevalence over the whole study period (40%), followed by IAV and HuCoVs, with prevalences of 18.4% and 12.8%, respectively. Several studies have shown that HRVs, due to their morphological characteristics, are highly resistant to environmental factors, and their circulation has not been affected by NPIs adopted to prevent SARS-CoV-2 infections. Furthermore, in vitro studies on human airway epithelial cells have shown that HRV replication is not impaired by concurrent SARS-CoV-2 infection. Conversely, primary HRV infection significantly reduces the replication of the Wuhan variant of SARS-CoV-2 through the induction of type I and III interferon responses. A similar, albeit less pronounced, inhibitory effect has been observed against the Delta and Omicron variants, confirming the ability of the more recent SARS-CoV-2 variants to evade host antiviral IFN responses [18,38,39].

The reduced virulence of the Omicron variant, combined with the “immune debt” in the population caused by limited exposure to common respiratory pathogens, may have contributed to a shift in the clinical landscape, allowing other viruses such as HRV to maintain their predominant role during and after the COVID-19 pandemic [40,41,42,43]. Although HRVs are generally responsible for mild infections, they can also be involved in asthma exacerbations, bronchiolitis, pneumonia, and cardiopulmonary diseases, proving to be a recognized cause of severe pneumonia [43,44,45,46]. However, 86% of HRV/EV-positive cases detected in Palermo (110/128) were not severe enough to require admission to ICUs. In contrast to HRV, most respiratory viruses, including PIV, ADV, RSV, HuBoV, MPV, and the influenza viruses, significantly decreased their incidence worldwide during the COVID-19 pandemic and showed a delayed, out-of-season resurgence with a higher median age of hospitalized patients following the relaxation of NPIs [15]. In fact, unusual influenza and RSV trends occurred worldwide throughout the COVID-19 pandemic and in the first post-pandemic season [15,21,47]. An earlier onset of IAV activity was observed in our study population during the 2022–2023 season, in accordance with the Italian National Epidemiological Surveillance System for influenza and other respiratory viruses (RESPIVIRNET) and the reports of the Global Influenza Surveillance and Response System (GISRS) [27,48]. In particular, among IAV infections in Palermo (which accounted for 90.8% of all influenza cases compared to 9.2% caused by influenza B), a prevalence of influenza A(H3N2) strains was demonstrated during the first post-COVID-19 pandemic season. This was subsequently replaced by the A(H1N1)pdm09 genotype, which peaked in circulation in December of the second season. Early onset of the influenza season in Sicily had been identified by environmental surveillance of virus RNA in wastewater as early as week 36/2022 and confirmed by syndromic sentinel surveillance in October 2022 [49,50]. Though RSV is commonly considered a typical agent of bronchiolitis in children, the recent literature indicates its relevant role also in severe infections in the elderly [51]. Accordingly, it was observed that 74.2% of RSV-positive patients belonged to the over-60 age group, underlining the role of this virus as a cause of hospitalization and severe outcomes in the elderly. Interestingly, RSV-B represented the prevalent genotype during the first post-COVID-19 pandemic season, being replaced by RSV-A in the following season. RSV epidemiology has been traditionally characterized by a poorly defined replacement of subgroups A and B, which has been related to the maintenance of its global circulation in the pre-pandemic period. However, in the post-COVID-19 pandemic period, a distinct RSV-B lineage concurrently spread in several countries, including Italy [52,53]. The emergence of this highly genetically divergent RSV-B lineage, together with declining population immunity, may have played a role in the sustained RSV circulation and the consistent impact on hospitalization [51,54]. In our study population of patients with severe acute respiratory symptoms, a viral etiology was most commonly found in NPS samples from patients admitted to non-intensive care units (Table 2). Only HuCoV positivity rates were higher in BAL samples compared to NPS samples (6.9% vs. 2.4%, *p* < 0.005). HuCoV-NL63, HuCoV-229E, HuCoV-HKU1, and HuCoV-OC43 are generally responsible for mild, self-limiting URTIs and common colds, but in our study, they followed HRV/EV as the second most frequent viruses in patients admitted to the ICU. The severity of acute respiratory infections requiring admission to the ICU is largely driven by a dysregulated inflammatory cascade, with non-coding RNAs emerging as key epigenetic regulators of immune gene expression. While these mechanisms are well characterized in COVID-19, their role in other infections remains poorly understood, yet is likely relevant for identifying novel biomarkers and therapeutic targets [55]. The role of HuCoVs as responsible for severe lower respiratory tract diseases, including pneumonia and bronchitis, has been demonstrated in high-risk populations, such as the elderly and immunocompromised individuals with pre-existing conditions [56]. Thanks to the increasing use of multiplex molecular assays in clinical settings, several serotypes of PIV, generally related to serious diseases and mortality in pediatric and immunocompromised populations, have more recently been linked to hospitalization in adults [57]. In our experience, PIV3 represented the most frequent genotype throughout the study period, being detected in 50% of PIV-positive patients, followed by PIV4, accounting for 20% of PIV-positive cases, mainly in December 2023. In the literature, most studies have documented PIV3 as a common cause of clinically significant infections, while PIV4 has been more rarely detected [57]. The increasing use of syndromic assays has also facilitated the detection of virus–virus and virus–bacteria/fungi multiple infections [58]. In our study, a wide range of virus–virus co-infections was detected in 10.9% of positive patients, mostly in upper respiratory samples (77.1%). Multiple infections involved almost all viruses detectable by the syndromic panels used. Several studies have reported that viral co-infections are common in the pediatric population and that the combinations of viruses may change depending on several factors, including host characteristics, viral seasonality, receptor competition/cell tropism, and viral interference, such as those that have been well documented between IAV and RSV [18,59]. Within our cohort, the virus most commonly detected in co-infections was HRV/EV, followed by ADV and PIV. The analysis of Ct values, a parameter widely used to estimate viral load, revealed a marked difference in Ct values in 53.3% of viral co-infections (13.6 average Ct difference between the viruses detected), allowing us to find the main pathogenic role of one of the two viruses involved. However, in 34.3% of cases, high Ct values (low viral load) were observed for both co-infecting viruses, making it difficult to attribute the etiological role to a specific pathogen. However, the role of viral co-infection in the severity of respiratory disease remains controversial, and the clinical relevance of multiple infections detected by molecular testing deserves further investigation. This study has several limitations. First, the absence of pre-pandemic data on the circulation of respiratory viruses in our geographical area prevented direct comparisons of viral dynamics before and after the pandemic. Second, the diagnostic approach relied on syndromic panels targeting a limited range of epidemiologically relevant pathogens; consequently, negative results cannot exclude infections caused by agents not included in the panels. Moreover, the use of different multiplex diagnostic tests could vary the performance of the diagnostic in terms of sensitivity and specificity, though the molecular tests we used demonstrated comparable performance in the literature (“Comparison of Biofire FilmArray Respiratory Panel, Seegene Anyplex II RV16, and Argene for the detection of respiratory viruses” by Chan M. et al.) [60]. Third, data on host-related factors, such as comorbidities and vaccination status, were not available, limiting our ability to evaluate their impact on disease severity. Finally, the study period coincided with the circulation of specific viral variants, which may affect the generalizability of our findings to other contexts of viral interaction.

## 5. Conclusions

This study demonstrates the sustained circulation of respiratory viruses in adults hospitalized with severe respiratory symptoms in a peculiar historical context, namely, the two seasons immediately following the COVID-19 pandemic. The routine use of syndromic panels proved to be an effective tool not only for diagnostic purposes, allowing for the early identification of viral infections and co-infections, but also for the valuable real-time tracking of the circulation of viral pathogens, including their main genotypes. The data generated by syndromic viral panels routinely used in hospital diagnostic microbiology laboratories can complement the epidemiological data from national and regional sentinel surveillance systems, serving as an early alert tool for both mild and severe respiratory infections requiring hospitalization. Importantly, our study contributes to expanding knowledge on respiratory illness in the adult population, which is often under-investigated in the context of viral respiratory infections, within a geographical setting characterized by intense migration and tourist flows. Nevertheless, we must acknowledge that the current diagnostic tools remain restricted to the detection of known and epidemiologically relevant viruses, which represent only a small fraction of the estimated human viruses [61]. In the near future, the growing application of next-generation sequencing technologies, capable of identifying a broader range of pathogens, is expected to further reshape the epidemiological landscape. Continued surveillance of respiratory viruses, along with strengthened interaction between clinicians and microbiologists, will be crucial to better characterizing the burden of viral respiratory infections, both in mono- and co-infections, and their impact on public health.

## Figures and Tables

**Table 1:** Correlations between positivity rate for viral infections and demographic information of hospitalized patients and hospitalization wards.

Variable	Number of Patients (%)	Virus-Positive Patients (%)	*p*-Value
Male	611 (56.5)	173 (54.1)	0.297
Age in years, median	71	69	
Age ranges (2022–2024)			
18–30 yrs	46 (4.3)	13 (28.3)	**0.014 ^a^**
31–40 yrs	41 (3.8)	19 (46.3)	
41–50 yrs	73 (6.8)	27 (37.0)	
51–60 yrs 61–70 yrs 71–80 yrs 81–90 yrs >91 yrs	151 (14.0)	46 (30.5)	
	219 (20.3)	71 (32.3)	
	336 (31.1)	75 (22.1)	
	194 (17.0)	62 (31.8)	
	21 (1.9)	7 (33.3)	
Admission ward			
ICU	242	49 (20.2)	**0.0003 ^a^**
Non-ICU	839	271 (32.3)	
Virus detection rate		320 (29.6)	0.701
Single virus detection		285 (26.4)	
Multiple virus detection		35 (3.2)	
Total	1081	320	

Note: Data are shown as mean or numbers (%). Abbreviations: yrs, years; ICU, intensive care unit; non-ICU, non-intensive care unit. **^a^** Bold character indicates statistical significance.

**Table 2:** Virus positivity rate in ICU and non-ICU inpatients and prevalence of viral pathogens in BAL and NPS.

Identified Virus	ICU (n = 242)	NON-ICU (n = 839)	*p*-Value ICU vs. NON-ICU	Total (n = 1081)	BAL (n = 318)	NPS (n = 792)	*p*-Value BAL vs. NPS	Total (n = 1110)
HRV/EV	18 (7.4)	110 (13.1)	**0.017 ^a^**	128 (11.8)	30 (9.4)	98 (12.4)	0.178	128 (11.5)
IAV	8 (3.3)	51 (6.1)	0.108	59 (5.4)	13 (4.1)	46 (5.8)	0.301	59 (5.3)
IBV	1 (0.4)	5 (0.6)	1.0	6 (0.6)	1 (0.3)	5 (0.6)	0.680	6 (0.5)
SARS-CoV-2	1 (0.4)	20 (2.4)	0.061	21 (1.9)	3 (0.9)	18 (2.3)	0.221	21 (1.9)
RSV	2 (0.8)	29 (3.4)	**0.028 ^a^**	31 (2.9)	6 (1.9)	25 (3.1)	0.315	31 (2.8)
MPV	3 (1.2)	18 (2.1)	0.596	21 (1.9)	6 (1.9)	15 (1.9)	1.00	21 (1.9)
ADV	1 (0.4)	22 (2.6)	**0.040 ^a^**	23 (2.1)	2 (0.6)	21 (2.6)	**0.035 ^a^**	23 (2.1)
PIV	3 (1.2)	23 (2.7)	0.236	26 (2.4)	5 (1.6)	21 (2.6)	0.389	26 (2.3)
HuCoVs	15 (6.2)	26 (3.1)	**0.035 ^a^**	41 (3.8)	22 (6.9)	19 (2.4)	**0.0006 ^a^**	41 (3.7)
HuBoV **^b^**	0	7 (0.9)	0.360	7 (0.9)	0	7 (1.0)	0.202	7 (0.9)

Note: ICU and non-ICU data are presented as the number (percentage) of patients, while BAL and NPS data are presented as the number (percentage) of samples. Abbreviations: HRV/EV rhinovirus/enterovirus; IAV, influenza A; IBV, influenza B; RSV, respiratory syncytial virus; HuCoVs, human coronaviruses (HuCoV NL63, HuCoV OC43, HuCoV 229E, and HuCoV-HKU1); HuBoV, human bocavirus; SARS-CoV-2, severe acute respiratory syndrome coronavirus 2; MPV, metapneumovirus; ADV, adenovirus; BAL, bronchoalveolar lavage; NPS, nasopharyngeal swab. ^a^ Bold character indicates statistical significance, calculated using Fisher’s exact test. ^b^ Human bocavirus (HuBoV) was not included in all syndromic panels used and was only tested in a subset of patients (n = 758) and a total of 763 samples (36 BAL and 727 NPS; 744 ICUs and 14 non-ICUs). Therefore, it was analyzed separately for descriptive purposes.

**Table 3:** Distribution of viral, non-viral, and mixed infections and negative respiratory samples by season, age, and sex.

Season	Viral Infections (%)		Non-Viral Infections (%)		Mixed Infections (%)		No Pathogens Detected (%)		Total	
2022–2023	42 (10.5)		156 (39.0)		61 (15.3)		141 (35.2)		400	
2023–2024	48 (11.1)		166 (38.2)		76 (17.5)		144 (33.2)		434	
Age ranges	M	F	M	F	M	F	M	F	M	F
18–30 yrs	2 (8.7)	1 (9.1)	14 (60.9)	3 (27.3)	2 (8.7)	4 (36.4)	5 (21.7)	3 (27.3)	23	11
31–40 yrs	3 (15.8)	4 (28.6)	8 (42.1)	2 (14.3)	2 (10.5)	5 (35.7)	6 (31.6)	3 (21.4)	19	14
41–50 yrs	3 (9.7)	4 (19.0)	9 (29.0)	11 (52.4)	11 (35.5)	1 (4.8)	8 (25.8)	5 (23.8)	31	21
51–60 yrs	8 (14.0)	7 (11.5)	19 (33.3)	24 (39.3)	7 (12.3)	13 (21.3)	23 (40.4)	17 (27.9)	57	61
61–70 yrs	17 (17.0)	13 (18.1)	39 (39.0)	22 (30.6)	16 (16.0)	10 (13.9)	29 (29.0)	27 (37.5)	100	72
71–80 yrs	20 (12.5)	12 (10.3)	71 (44.4)	51 (43.6)	12 (7.5)	14 (12.0)	57 (35.6)	40 (34.2)	160	117
81–90 yrs	11 (14.7)	8 (13.1)	28 (37.3)	16 (26.2)	8 (10.7)	7 (11.5)	28 (37.3)	30 (49.2)	75	61
>91 yrs	2 (20.0)	0 (0.0)	4 (40.0)	1 (50.0)	0 (0.0)	1 (50.0)	4 (40.0)	0 (0.0)	10	2

Note: Data are shown as numbers and percentages. Abbreviations: yrs, years; M, male; F, female.

## Data Availability

All data analyzed in this study are included in the article and its Supplementary Materials. Further inquiries can be directed to the corresponding author.

## Supplementary Materials

The following supporting information can be downloaded at https://www.mdpi.com/article/10.3390/pathogens14100997/s1: Figure S1: Study population and inclusion criteria of the analysis. Table S1: Syndromic assays used on NPS and BAL samples required by ICUs and non-ICUs. Table S2: Prevalence of viral infections in different age groups. Table S3: Ct values in virus associations of 35 co-infections. Table S4: Detection of viral targets in mono-infections or co-infections.

## Abbreviations

The following abbreviations are used in this manuscript:

RTI	Respiratory tract infection
URTIs	Upper respiratory tract infections
LRTIs	Lower respiratory tract infections
COPD	Chronic obstructive pulmonary disease
IAV	Influenza A virus
IBV	Influenza B virus
RSV	Respiratory syncytial virus
PIVS	Parainfluenza viruses 1/2/3/4
ADV	Adenovirus
SARS-CoV-2 or COVID-19	Severe acute respiratory syndrome coronavirus 2
NPIs	Non-pharmaceutical interventions
NPS	Nasopharyngeal swab or nasal swab
BAL	Bronchoalveolar lavage or sputum
ICU	Intensive care unit
non-ICU	Non-intensive care unit
FAPP	FilmArray Pneumonia plus
RP2.1	FilmArray Respiratory Panel 2.1
Ct	Threshold cycle
HRV/EV	Human rhinovirus/enterovirus
HuCoVs	Human coronaviruses (HuCoV-NL63, HuCoV-OC43, HuCoV-229E, and HuCoV-HKU1)
HuBoV	Human bocavirus
FLU	Influenza virus
MPV	Metapneumovirus
CAP	Community-acquired pneumonia
HAP	Hospital-acquired pneumonia
PCR	Polymerase Chain Reaction
IQR	Interquartile range
