# Supplementary material for: Post-COVID-19 Epidemiology of Viral Infections in Adults Hospitalized with Acute Respiratory Syndromes in Palermo, South of Italy
Source: Pathogens. 2025 Oct 2;14(10):997. doi: 10.3390/pathogens14100997 (PMC12567287; doi:10.3390/pathogens14100997)
Supplement: Supplementary file 1 [file pathogens-14-00997-s001.zip › pathogens-3801584-supplementary.pdf]

pathogens MDPI

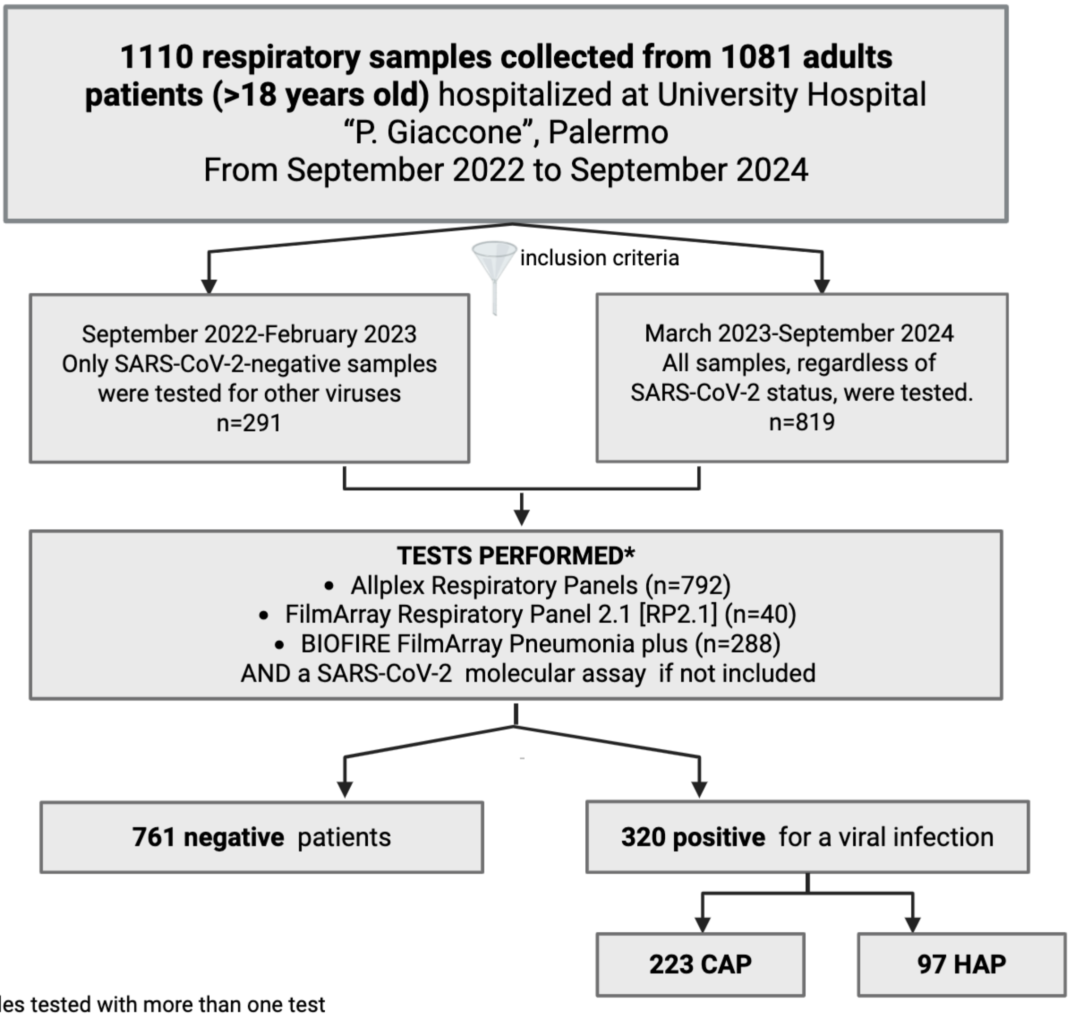

- 10 samples tested with more than one test

Figure S1. Study population and inclusion criteria of the analysis.

Table S1. Syndromic assays used on NPS and BAL samples ordered by ICU and Non-ICU.

Syndromic kit	Units	BAL (%)*	NPS (%)*
BIOFIRE FilmArray Pneumonia plus [FAPP]	ICU	229 (70.7)	-
	non-ICU	56 (17.3)	3 (0.4) **
Respiratory Panel 2.1 [RP2.1]	ICU	1(0.3)**	2 (0.3)
	non-ICU	-	37 (4.6)
RVMaster assay and Allplex Respiratory Panels 1-3	ICU	9 (2.8)	9 (1.1)
	non-ICU	29 (9.0)	745(93.6)

*Percentage calculated on the total number of assays performed on BAL (n=324) and NPS samples (n=796). ICU, intensive care units; non-ICU, non- intensive care units; BAL, bronchoalveolar lavage; NPS, nasopharyngeal swab. ** Test performed off-label for non-validated matrices according to the manufacturer's instructions, based on specific clinical needs and sample availability.

Table S2. Prevalence of viral infections in different age groups.

Virus/Age	n. positive (%)	*p*-value
HRV/EV	128 (11.8)	<0.05
18-30 years	8 (17.4)	0.237
31-40 years	12 (29.3)	**0.017**[a]
41-50 years	8 (11)	1.00
51-60 years	19(12.6)	0.785
61-70 years	27 (12.3)	0.815
71-80 years	30 (8.8)	**0.043**
81-90 years	21(10.8)	0.713
> 90 years	3 (14.3)	0.729
IAV	59 (5.53)	>0.05
18-30 years	2 (4.3)	1.00
31-40 years	2 (4.9)	1.00
41-50 years	7 (9.6)	0.109
51-60 years	11 (7.3)	0.331
61-70 years	9 (4.1)	0.406
71-80 years	14 (4.1)	0.248
81-90 years	13 (6.7)	0.386
> 90 years	1 (4.8)	1.00
IBV	6 (0.6)	<0.05
18-30 years	2 (4.3)	**0.024**
31-40 years	0 (0.0)	1.00
41-50 years	2 (2,7)	0.056
51-60 years	0 (0.0)	1.00
61-70 years	1 (0.5)	1.00
71-80 years	1(0.3)	0.672
81-90 years	0 (0.0)	0.598
> 90 years	0 (0.0)	1.00
SARS-CoV-2	21 (1.93)	>0.05
18-30 years	1 (2.17)	0.601
31-40 years	0 (0.0)	1.00

41-50 years	2 (2.74)	0.647
51-60 years	2 (1.32)	0.756
61-70 years	5 (2.27)	0.595
71-80 years	3 (0.88)	0.101
81-90 years	7 (3.59)	0.081
> 90 years	1 (4.76)	0.339
RSV	31 (2.85)	>0.05
18-30 years	0 (0.0)	0.638
31-40 years	2 (4.9)	0.328
41-50 years	2 (2.7)	1.00
51-60 years	4 (2.6)	1.00
61-70 years	10 (4.5)	0.110
71-80 years	6 (1.8)	0.172
81-90 years	6 (3.1)	0.813
> 90 years	1 (4.8)	0.459
MPV	21 (1.93)	>0.05
18-30 years	1 (2.2)	0.601
31-40 years	0 (0.0)	1.00
41-50 years	2 (2.7)	0.647
51-60 years	2 (1.3)	0.756
61-70 years	5 (2.3)	0.595
71-80 years	6 (1.8)	1.00
81-90 years	5 (2.6)	0.563
> 90 years	0 (0.0)	1.00
ADV	23 (2.11)	>0.05
18-30 years	0 (0.0)	0.602
31-40 years	3 (7.3)	0.052
41-50 years	2 (2.7)	0.664
51-60 years	2 (1.3)	0.759
61-70 years	5 (2.3)	0.796
71-80 years	7 (2.1)	1.00
81-90 years	4 (2.1)	1.00
> 90 years	0 (0.0)	1.00
PIV	26 (2.4)	>0.05
18-30 years	2 (4.3)	0.302
31-40 years	2 (4.9)	0.257
41-50 years	1 (1.4)	1.00
51-60 years	1 (0.7)	0.244
61-70 years	6 (2.7)	0.804
71-80 years	4 (1.2)	0.088
81-90 years	10 (5.1)	**0.016**
> 90 years	0 (0.0)	1.00
HuCoV	41 (11.0)	>0.05

18-30 years	0 (0.0)	0.413
31-40 years	1 (2.4)	1.0
41-50 years	5 (6.8)	0.189
51-60 years	6 (4.0)	0.820
61-70 years	12 (5.5)	0.164
71-80 years	13 (3.8)	1.0
81-90 years	4 (2.1)	0.213
> 90 years	0 (0.0)	1.0
HuBoV[b]	7 (0.9)	>0.05
18-30 years	0 (0.0)	1.0
31-40 years	0 (0.0)	1.0
41-50 years	0 (0.0)	1.0
51-60 years	3 (2.9)	0.058
61-70 years	2 (1.4)	0.618
71-80 years	0 (0.0)	0.113
81-90 years	1(0.6)	1.0
> 90 years	1 (5.3)	0.170

Note: The percentage is calculated as the ratio of virus-positive patients to the total number of patients screened in that age group. Abbreviations: HRV/EV rhinovirus/enterovirus, IAV influenza A, IBV influenzaB, RSV respiratory syncytial virus, HuCoV human coronavirus , HuBoV human bocavirus , SARS-CoV-2 severe acute respiratory syndrome coronavirus 2, MPV metapneumovirus, ADV adenovirus. [a]Bold character indicates statistical significance; [b]HuBoV was tested on 757 patients, as not all molecular panels applied included this virus. Statistical comparisons were performed separately on the subgroup of patients tested.

Table S3. Ct values in virus associations of 35 co-infections.

Virus 1	Ct	Virus 2	Ct	Virus 3	Ct
HRV/EV	34.4	PIV	35		
IAV	22.5	HRV/EV	40.7		
HuCoV	16	IAV	37		
IAV	22.3	HRV/EV	38.8		
MPV	22	HRV/EV	35		
IBV	31	IAV	35	MPV	>40
HuBoV	30.8	HRV/EV	31.6		
HRV/EV	29.5	ADV	32.7		
SARS-CoV-2	33.4	HRV/EV	34.3		
HRV/EV	37.3	ADV	39.5		
IAV	22.9	PIV	36.6		
PIV	36.8	ADV	38.6		
PIV	34.5	HRV/EV	36.2		
HRV/EV	27.9	ADV	38.3		
PIV	35.2	HRV/EV	35.9	ADV	36.2
HRV/EV	27.2	HuCoV	40.1		
SARS-CoV-2	ND	HRV/EV	ND		
RSV-B	40.5	HuBoV	40.6		
IAV	23.4	ADV	36.9	HRV/EV	37.3
ADV	37.8	IAV	37.9		
HuCoV	21.7	HRV/EV	28.9		
RSV-A	22	HuCoV	34		
HuCoV	12.2	ADV	32.8	RSV-A	33.12
HuCoV	19.5	HuBoV	38.6		
HRV/EV	29.3	MPV	36.4		

HuCoV	18.7	HuBoV	29.6		
ADV	25.2	HRV/EV	38.9		
PIV	31.6	ADV	37.3	HRV/EV	>40
HRV/EV	33.6	MPV	38.1		
MPV	27.5	PIV	38.6		
HuCoV	25	PIV	38	HRV/EV	>40
PIV	18	MPV	38		
SARS-CoV-2	ND	IAV	ND		
IAV	ND	SARS-CoV-2	ND		
PIV	ND	HRV/EV	ND		

Abbreviations: HRV/EV rhinovirus/enterovirus, IAV influenza A, IBV influenza B, RSV-A respiratory syncytial virus A, RSV-B respiratory syncytial virus A HuCoV human coronavirus (HuCoV NL63, HuCoV OC43, HuCoV 229E) HuBoV human bocavirus , SARS-CoV-2 severe acute respiratory syndrome coronavirus 2, MPV metapneumovirus, ADV adenovirus, PIV parainfluenza viruses, Ct threshold cycle, ND not determined.

Table S4. Detection of viral targets in mono-infections or co-infections.

Virus detection	Mono-infections	Co-infections	*p*-value [a]
HRV/EV (N=128)	107 (83.6)	21 (16.4)	**<0.001**
IAV (N=59)	51 (86.4)	8 (13.6)	**<0.001**
IBV (N=6)	5 (83.3)	1 (16.7)	0.218
SARS-CoV-2 (N=21)	17 (81.0)	4 (19.0)	**0.007**
RSV (N=31)	28 (90.3)	3 (9.7)	**<0.001**
MPV (N=21)	15 (71.4)	6 (28.6)	0.078
ADV (N=23)	13 (56.5)	10 (43.5)	0.678
PIV (N=26)	16 (61.5)	10 (38.5)	0.327
HuCoV (N=41)	32 (78.0)	9 (22.0)	**<0.001**
HuBoV (N=7)	3(42.9)	4 (57.1)	1

Note: Data are presented as the number (percentage) of patients. Abbreviations: HRV/EV rhinovirus/enterovirus, IAV influenza A, IBV influenza B, RSV respiratory syncytial virus, HuCoV human coronavirus, HBoV human bocavirus, SARS-CoV-2 severe acute respiratory syndrome coronavirus 2, MPV metapneumovirus, ADV adenovirus, EV enterovirus. [a] Bold character indicates statistical significance, p-values were calculated by binomial test.
